# HPV infection and bacterial microbiota in the placenta, uterine cervix and oral mucosa

**DOI:** 10.1038/s41598-018-27980-3

**Published:** 2018-06-28

**Authors:** Heidi Tuominen, Samuli Rautava, Stina Syrjänen, Maria Carmen Collado, Jaana Rautava

**Affiliations:** 1 Department of Oral Pathology and Oral Radiology, Institute of Dentistry, Faculty of Medicine, University of Turku, Turku, Finland; 2 Department of Paediatrics, University of Turku & Turku University Hospital, Turku, Finland; 3 Department of Pathology, Turku University Hospital, Turku, Finland; 4 Department of Biotechnology, Institute of Agrochemistry and Food Science, Spanish National Research Council (IATA-CSIC), Valencia, Spain

## Abstract

We investigated the association between HPV infection and bacterial microbiota composition in the placenta, uterine cervix and mouth in thirty-nine women. HPV DNA genotyping of 24 types was conducted using Multimetrix^®^. Microbiota composition was characterized by 16S rRNA gene sequencing. HPV DNA was detected in 33% of placenta, 23% cervical and 33% oral samples. HPV16 was the most frequent type in all regions. HPV infection was associated with higher microbiota richness (p = 0.032) in the mouth but did not influence microbial diversity or richness in other samples. HPV infection was associated with higher abundance of *Lactobacillaceae* (p = 0.0036) and *Ureaplasma* (LDA score > 4.0, p < 0.05) in the placenta, *Haemophilus* (p = 0.00058) and *Peptostreptococcus* (p = 0.0069) genus in the cervix and *Selenomonas* spp. (p = 0.0032) in the mouth compared to HPV negative samples. These data suggest altered bacterial microbiota composition in HPV positive placenta, cervix and mouth. Whether the changes in bacterial microbiota predispose or result from HPV remains to be determined in future studies.

## Introduction

Human papilloma virus (HPV) is a known oncovirus and it has potential to cause carcinoma in the genital, anal and oropharyngeal regions^1^. HPV has long been regarded mainly as a sexually transmitted disease (STD) but recent studies have shown that HPV infection can be acquired by vertical transmission as well as via the placenta from mother to child^2–7^. Traditionally, HPV infection in the placenta has been considered to increase the risk of pregnancy complications^8–11^ but HPV DNA has also been discovered from placentas from healthy pregnancies^3,5,12–14^ and transabdominally obtained placental samples^7,15,16^.

In addition to viruses, humans have diverse bacterial microbiota which has been recognized to play an important role in human health. Recently published data suggest that even the placenta may harbor a unique microbiota which is mainly composed of *Proteobacteria*^17,18^. Parnell *et al*.^19^ have reported that the microbiota composition varies depending on the location within the placenta and that the placental microbiota may change according to maternal factors such as gestational diabetes mellitus^20^. However, not all published data support the notion of intrauterine colonization^21,22^.

Healthy vaginal microbiota is known to be predominantly dominated by lactobacilli. Shifts in vaginal microbiota balance may result in altered composition referred to as polybacterial dysbiosis and to disease such as bacterial vaginosis, both of which have been connected to vaginal HPV infection^23,24^. Individuals with HPV infection more frequently have higher diversity of cervico-vaginal microbiota and HPV clearance in turn has been associated with increased number of antigen-representing Langerhans cells^25^. A study by Gao *et al*.^26^ showed more *L*. *gasseri* and *Gardnerella vaginalis* in HPV infected women’s cervico-vaginal microbiota. HPV persistence is considered a critical event in malignant transformation. We have previously shown that bacterial vaginosis, even when asymptomatic, predicted HPV persistence^23^. Di Paola *et al*.^27^ propose that *Atopobium* spp. and *G*. *vaginalis* may serve as microbial markers for HPV persistence in Italian women while Adebamowo *et al*.^28^ showed that *Mycoplasma hominis* was associated with HPV persistence in Nigerian women. Furthermore, changes in the vaginal microbial composition, connected to lower levels of *Lactobacillus* spp., have been associated with more severe cervical intraepithelial neoplasia and higher diversity in microbiota might lead to more severe disease^29^.

Recent research has revealed that malignancies may arise in various human organs in reaction to bacterial inflammation or by bacteria’s own direct oncogenic mutations to human cells. Bacteria have been linked with conditions including liver cancer, esophageal cancer, pancreatic cancer, gall bladder cancer and colorectal cancer^30,31^. Furthermore, there are differences in the microbiota between normal oral mucosa and patients with oral squamous cell carcinoma^32^. It has even been suggested that the oral microbiota may have an effect on developing pancreatic cancer, possibly mediated by *P*. *gingivalis*. *P*. *gingivalis* has also been linked to chronic inflammation and atherosclerosis^33,34^. Accumulating evidence thus suggests that both HPV and bacterial dysbiosis might play a significant role in malignant transformation. Nonetheless, our knowledge about the interactions between HPV infection and the bacterial microbiota and its impact on human health is still rudimentary.

We aimed to investigate whether an existing HPV infection has influence on the bacterial microbiota composition in the placenta, the uterine cervix or the mouth. This could lead us to understand part of the mechanisms modifying HPV clearance and persistence leading to malignancy.

## Results

### HPV status of the samples

The HPV genotypes of all the samples including sex of the infant, delivery mode and gestational age are presented in Table 1. HPV DNA was detected in 33% of the placental, 23% cervical and 36% oral samples. HPV16 was the most frequent type found in all groups studied (54% of placenta, 22% of cervix and 54% of oral samples). Infection with multiple HPV types was found in three oral and five cervical samples but in none of the placental samples. Four women displayed HPV DNA both in the oral and cervical samples, seven in oral and placental samples and six in cervical and placental samples. Of these, two women in oral/cervix, two in oral/placenta and four in cervix/placenta samples had the same HPV genotype in the two different sites, either HPV6 or HPV16. Only two women had HPV detectable in all three locations. Interestingly, the HPV genotype found was the same in all sites, i.e. either HPV6 or HPV16.

**Table 1:** HPV status of the samples (mother’s oral and cervical samples were collected during the last trimester).

No	case 1/control 2	Infant sex (1 boy, 2 girl)	Delivery mode (1 vaginal, 2 cs)	Gestation weeks	Mother oral (HPV type)	Mother cervix (HPV type)	Placenta (HPV type)
1	2	2	1	42.0	16	6,11,16,66	
2	1	2	1	41.0	66		16
3	2	2	2	35.4			
4	2	2	1	40.1			
5	2	1	1	37.6			
6	2	2	1	40.1	16		
7	2	1	1	42.0	16	16,33	
8	2	2	1	39.4			
9	2	2	1	38.3			
10*	2	1	1	38.0			
11*	2	1	1	38.0			
12	2	1	2	38.5			
13	2	1	2	42.2			
14	1	2	1	41.5	16,18		16
15	2	1	1	41.0			
16	2	1	1	39.0			
17	2	2	2	40.0	16		
18	2	1	2	42.1			
19	1	2	2	42.4			6
20	2	2	2	41.1		16,31,66	
21	2	2	1	39.6			
22	2	1	2	38.4			
23	1	2	1	39.0			16
24	1	1	1	42.0		6,59	6
25	1	2	1	40.4	6,16,18		16
26	2	2	2	39.0			
27	1	1	1	40.4	11		83
28	1	2	1	41.0	6,16		16
29	2	2	2	39.1			
30	1	2	1	41.6	16	16	16
31	2	2	2	40.6			
32	2	1	1	38.3			
33	1	2	1	40.6	6	6	6
34	2	2	1	40.0			
35	2	2	1	39.0			
36	2	2	2	41.4	16		
37	1	1	1	39.6		6,16,42,56	6
38	2	1	2	35.3	16		
39	1	1	1	36.6		6	6
40	1	1	2	42.0		16	16

^*^Twins.

### Placenta microbiota and HPV

**Figure 1:**
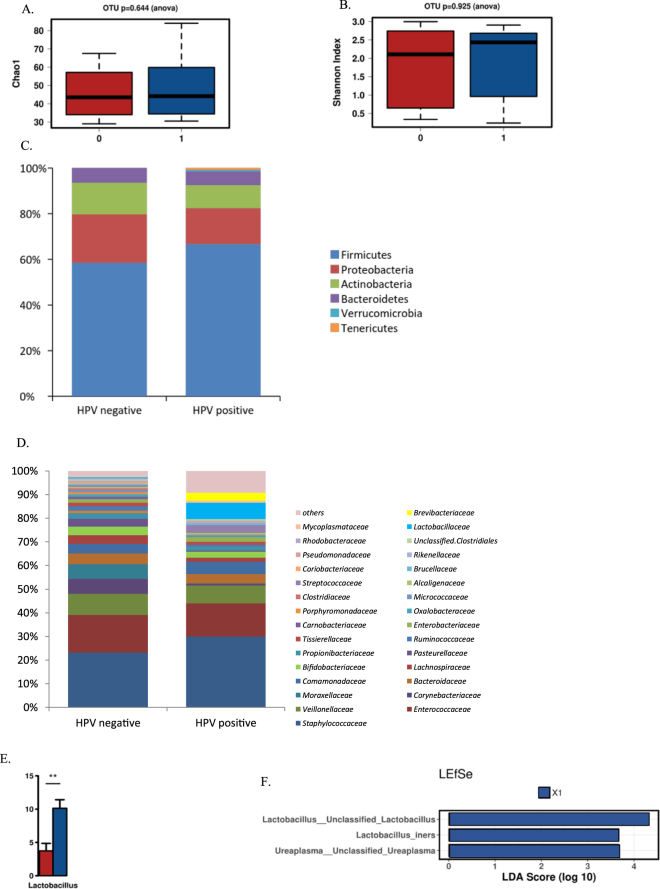
The bacterial microbiota in placenta samples negative and positive for HPV DNA. No significant differences in microbial richness (**A**) or diversity (**B**) were detected between HPV negative (red) and positive (blue) placenta samples. The relative abundance of bacteria are presented on the phylum (**C**) and family levels (**D**). The relative abundance of the genus *Lactobacillus* was significantly higher in HPV positive (blue) as compared to HPV negative (red) samples (**E**). By LEfSe analysis (**F**), the genera *Lactobacillus* and *Ureaplasma* were significantly enriched in HPV positive (blue) placenta samples as compared to HPV negative (LDA score > 4.0, p > 0.05).

To study the impact of potential contaminants, pooled placental negative controls yielded 726 reads and the identified OTUs (n = 11) representing 6 different genera listed in Supplementary Table 1. Finally, a total of 19/39 placenta samples (49%) were included in the analysis after exclusion of the specimens with low number of reads (n < 1000 reads) followed by filtering the OTUs present in the negative controls. The most abundant phylum in the placenta microbiota was *Firmicutes* (58.3% vs. 66.6%) followed by *Proteobacteria* (21.1% vs. 15.7%), *Actinobacteria* (13.8% vs 10.0%) and *Bacteroidetes* (6.5% vs. 6.1%) in HPV negative and positive respectively (Fig. 1C). At family level, *Staphylococcaceae* (22.5% vs 29.1%), *Enterococacceae* (15.6% vs 13.8%), *Veillonellaceae* (8.8% vs 7.4%), *Corynebacteriaceae* (6.3% vs 0.8%) and *Moraxellaceae* (6.1% vs 0.1%) were the predominant groups in both HPV negative and positive samples, respectively. At genus level, *Staphylococcus* (22.9% vs 29.5%); unclassified *Enterococcaceae* genus (15.6% vs 13.7%), *Corynebacterium* (6.3% vs 0.1%), and *Acinetobacter* (6.0% vs 0.1%) were the most abundant in HPV negative and positive placenta samples.

No differences in bacterial richness or diversity (as assessed by the Chao1 and Shannon indexes, respectively) were found between HPV positive (n = 6) and negative (n = 13) placenta groups (Fig. 1A,B). However, higher abundance of *Lactobacillaceae* (p = 0.0036) and *Lactobacillus* genus (p = 0.0023), were observed in HPV positive group compared to HPV negative placenta samples (Fig. 1,E). Interestingly, the relative abundance of *Lactobacillus iners* was higher in HPV negative group than in HPV positive (47.7% vs. 18.7% and p = 0.07). We also performed detailed analyses of the *Lactobacillus* species associated with HPV positivity in the placenta samples. In the HPV positive group, we detected *L*. *zeae*, *L*. *reuteri* and Unclassified *Lactobacillus*. To further investigate the composition, we selected LEfSe analyses at OTU level to reveal the *Lactobacillus* group in HPV positive placenta samples to be combined by *L*. *iners*, *L*. *crispatus*, *L*. *jensenii*, *L*. *gasseri and L*. *reuteri*. In addition, in HPV negative group *L*.*iners* was evident. LEfSe analysis showed that *Ureaplasma* and *Lactobacillus* genus were significantly enriched in HPV positive placenta samples compared to HPV negative (LDA score > 4.0, p < 0.05), (Fig. 1F).

Despite the lower number of HPV positive samples, comparing HPV positive samples between low-risk HPV types (LR-HPV, including HPV6, 11, 42, 43, 44 and 70) and high-risk HPV types (HR-HPV, including HPV16, 18, 26, 31, 33, 35, 39, 45, 51, 52, 53, 56, 58, 59, 66, 68, 73 and 82) genotypes we observed higher abundance of *Staphylococcus* (p = 0.012) and *Lachnospira* genus (p = 0.050) in LR-HPV genotype compared to those observed in HR-HPV genotype. Although not with significant statistical differences, a higher diversity and lower richness were observed in HR-HPV genotype compared to LR-HPV genotype. LEfSe analysis showed that *Ureaplasma* and *Lactobacillus* genus were significantly enriched in HR-HPV genotype placenta samples while *Lachnospira* genus was enriched in LR-HPV genotype.

### Cervical microbiota and HPV

**Figure 2:**
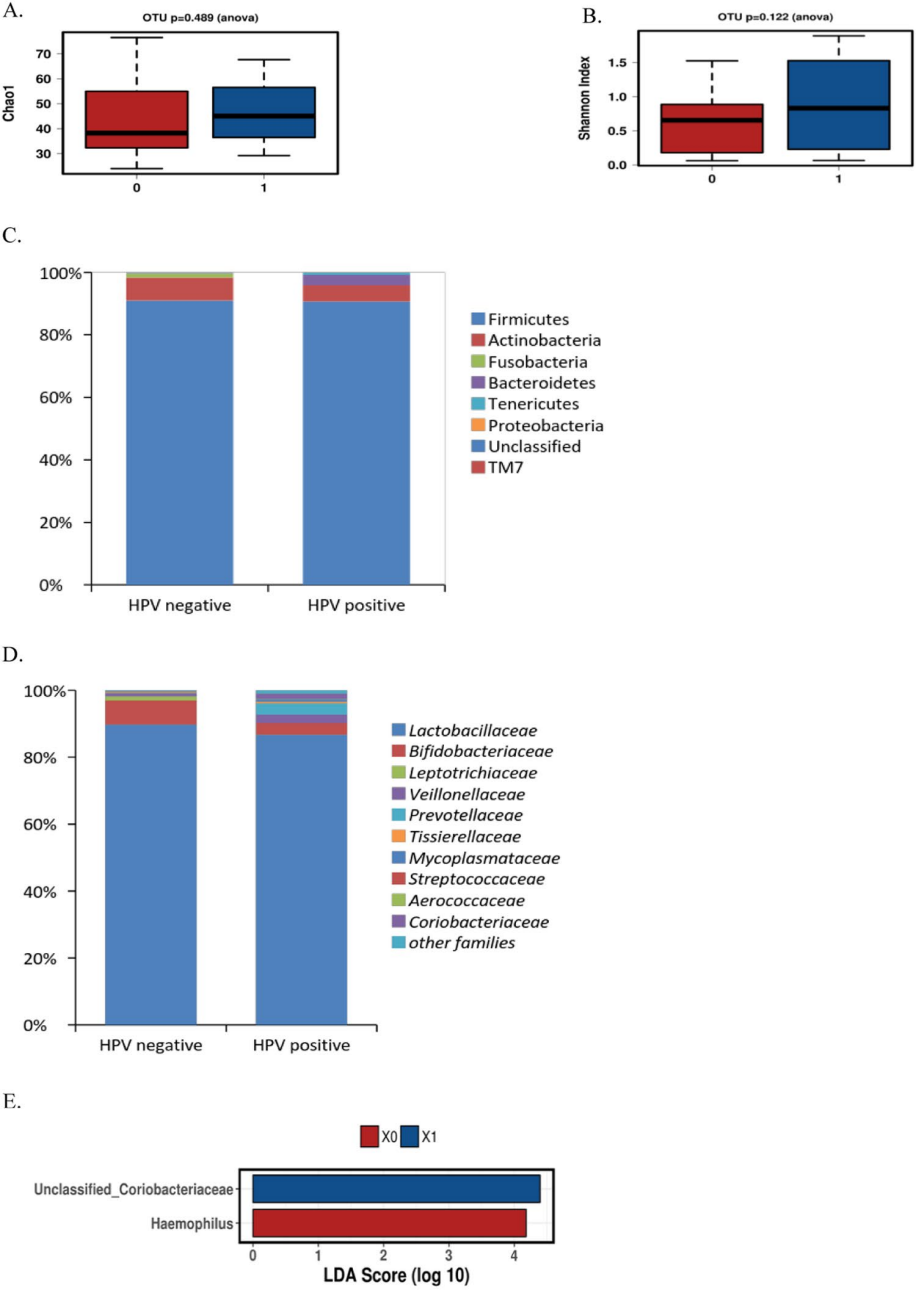
The bacterial microbiota in cervical samples negative and positive for HPV DNA. No significant differences in microbial richness (**A**) or diversity (**B**) were detected between HPV negative (red) and positive (blue) cervical samples. The relative abundance of bacteria are presented on the phylum (**C**) and family levels (**D**). By LEfSe analysis (**E**), unclassified *Coriobacteriaceae* were significantly enriched in HPV positive (blue) samples as compared to HPV negative (LDA score > 4.40, p < 0.05), while *Haemophilus* (LDA score > 4.18) were enriched in HPV negative (red) compared to HPV positive cervical specimens.

The most abundant phyla in the cervix were *Firmicutes* (91.0% vs. 90.7%) and *Actinobacteria* (7.3% vs. 5.1%), followed by *Fusobacteria* (1.2% vs. 0.1%), *Bacteroidetes* (0.2% vs. 3.3%) and *Tenericutes* and *Proteobacteria* in HPV negative and positive samples, respectively. No statistically significant differences in species richness or diversity were detected between HPV positive and negative cervical samples (Fig. 2A,B) nor were we able to point out statistical differences on the phylum level (Fig. 2C).

On the family level, the most abundant groups belonged to *Lactobacillaceae* (89.7% vs. 86.8% in HPV negative and positive samples, respectively). The HPV positive cervical samples mainly harboured *L*. *crispatus* and *L*. *jensenii* whereas in HPV negative cervix group *L*. *iners* and *L*. *reuteri* were more abundant. The relative abundance of *Coriobacteriaceae* (p = 0.083) was slightly increased in HPV positive cervical samples. On the other hand, *Peptostreptococcaceae* (p = 0.0065) and *Enterococcaceae* (p = 0.022) families were significantly increased in HPV negative compared to HPV positive cervical samples (Fig. 2D). The HPV positive cervix harbored more unclassified *Coriobacteriaceae* (p = 0.07) genus as compared to HPV negative cervical samples whereas *Haemophilus* (p = 0.00058) and *Peptostreptococcus* (p = 0.0069) were significantly increased in HPV negative group at genus level. At genus level, *Lactobacillus* was predominant in both HPV groups; negative and positive (89.7% in HPV negative and 86.7% in HPV positive). At species level, *L*. *iners*, mainly the OTU 133075, was the predominant *Lactobacillus* group in both HPV negative and positive samples (47.7% vs 18.6%, respectively, p-value = 0.07).

In LEfSe analysis, unclassified *Coriobacteriaceae* genus was significantly enriched in HPV positive group compared to HPV negative (LDA score > 4.40, p < 0.05), while *Haemophilus* (LDA score > 4.18) were enriched in HPV negative compared to HPV positive cervix (Fig. 2E). To further identify the species abundant in the HPV positive cervical samples group, we discovered the group mainly being composed of *Atopobium vaginae*.

### Oral microbiota and HPV

The most abundant phyla in the mouth were *Firmicutes* (48.9% vs. 53.5%), *Proteobacteria* (24.6% vs. 18.6%), *Actinobacteria* (12.5% vs. 11.8%), *Bacteroides* (8.7% vs. 10.6%) followed by *Fusobacteria* (3.8% vs. 4.5%) and *TM7* (0.6% vs. 1.0%) in HPV negative and positive samples, respectively. The predominant microbial families belonged to *Streptococcaceae*, *Pasteurellaceae*, *Veillonellaceae*, *Micrococcaceae*, *Prevotellaceae*, *Neisseriaceae*, *Gemellaceae* and *Fusobacteriaceae*. At genus level, *Streptococcus*, *Haemophilus*, *Veillonella*, *Prevotella*, unclassified *Gemellaceae*, *Fusobacterium*and *Actinomyces* were the predominant bacterial genera in both groups.

At phylum level, the relative abundance of *TM7* was increased (p = 0.026) in HPV positive oral samples compared to the HPV negative group by ANOVA test. Increased relative abundance of *TM73* (p = 0.011) at family level as well as *Selenomonas* spp. (p = 0.0032), *Megasphaera* spp. (p = 0.026) and TM73 (p = 0.018) at species level was detected in HPV positive oral samples. *Haemophilus* spp. was higher (p = 0.019) in HPV negative compared to HPV positive.

**Figure 3:**
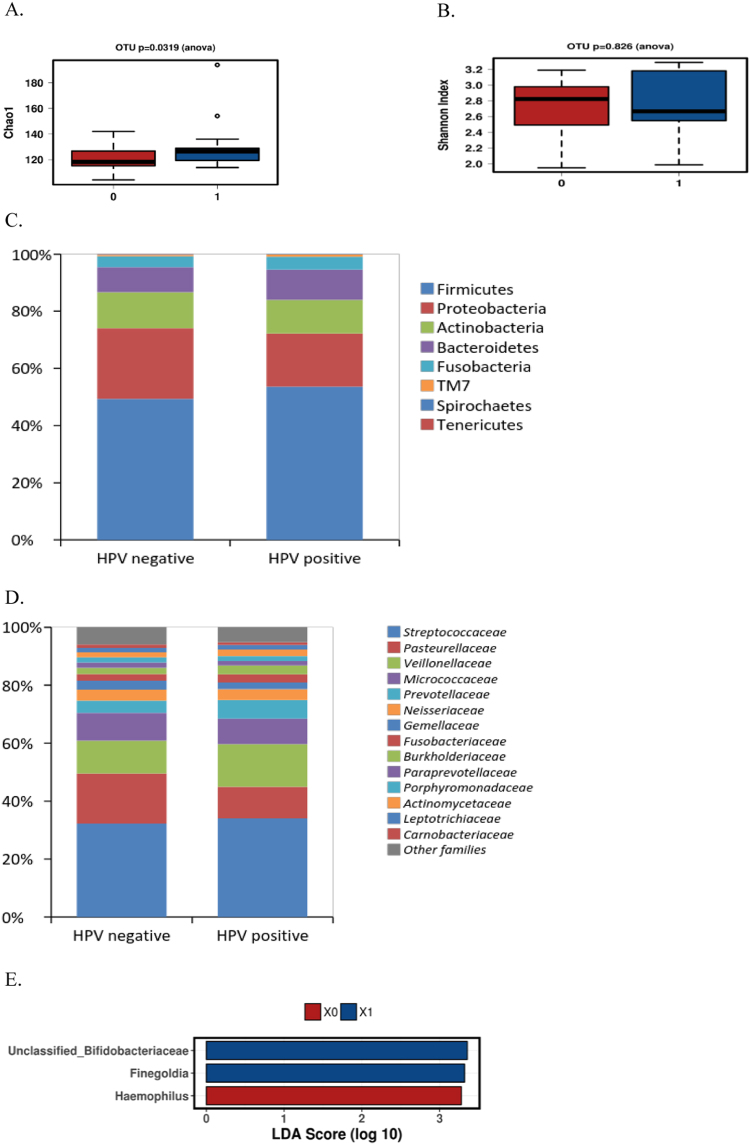
The bacterial microbiota in oral samples negative and positive for HPV DNA. Increased bacterial richness as assessed by the Chao1 index (**A**) was detected in HPV positive oral samples (blue) as compared to HPV negative samples (red). No difference in bacterial diversity (**B**) was detected between HPV negative (red) and positive (blue) oral samples. The relative abundance of bacteria are presented on the phylum (**C**) and family levels (**D**). In LEfSe analysis, unclassified *Bifidobacteriaceae* and *Finegoldia* genera were significantly enriched in HPV positive (blue) samples as compared to HPV negative (red) (LDA score > 3.36 and 3.32 respectively, p < 0.05), while *Haemophilus* genus was enriched in HPV negative (red) compared to HPV positive (**E**).

HPV positive samples displayed higher richness (Chao1 index) of bacterial microbiota as compared to HPV negative samples (p = 0.0319) (Fig. 3A). No difference in diversity (Shannon index, Fig. 3B) was detected between HPV positive and HPV negative groups. Furthermore, the LEfSe algorithm showed that unclassified *Bifidobacteriaceae* and *Finegoldia* genera were significantly enriched in HPV positive samples as compared to HPV negative (LDA score > 3.36 and 3.32 respectively, p < 0.05), while *Haemophilus* genus was enriched in HPV negative compared to HPV positive (Fig. 3E).

## Discussion

HPV infection is associated with altered bacterial microbiota in the placenta, uterine cervix and mouth. Distinct differences were detected in the relative abundance of specific microbial groups on family and genus levels while the overall microbial richness and diversity appeared to be associated with HPV infection with the exception of higher richness in HPV positive oral samples compared to HPV negative samples. HPV positive placenta samples showed increased abundance of *Lactobacillus* genus (p = 0.0023) and *Ureaplasma* (LDA score > 4.0, p < 0.05), *Coriobacteriaceae* (p = 0.083) in cervix and *Selenomonas* spp. (p = 0.0032), *Megasphaera* spp. (p = 0.026) and *TM73* (p = 0.018) in the mouth. In cervical samples *Peptostreptococcaceae* (p = 0.0065) and *Enterococcaceae* (p = 0.0069) were significantly enriched in HPV negative samples.

It has recently been suggested that the fetus does not develop in a sterile environment based on discovery of a distinct microbiota in the placenta^17,18^. Still there has been controversy about whether the findings are genuine or a result from contamination^21,35^. Some studies have not found specific placental microbiota^21,22^. Several studies have also reported HPV DNA in the placenta, even in samples collected transabdominally and sometimes associated with adverse effects in pregnancies^3,5,8–15,36,37^.

In this study we attempted to meticulously rule out the possibility of false positive results of either with bacterial microbiota or HPV DNA. We took special care and performed several steps to ensure that our findings are relevant and not result from contamination. The reagents used for PCR amplification were sequenced as well as negative controls to rule out possible reagent contamination. With placenta samples, negative placental controls reached 726 reads and identified 11 OTUs. We considered >1000 reads as a limit for inclusion. A total of 19/39 placenta samples (49%) reached that and therefore were included in this study. At OTU picking we further excluded singletons and OTUs with a relative frequency below 0.01. *Cyanobacteria*, *Chloroplasts* and *Rhizobiales* and sequences that could not be classified to domain level at OTU picking were removed from the results to control any possible bias. The presence and localization of HPV infection of our placental samples (Finnish Family HPV Study samples) have been examined before by Sarkola *et al*.^12^ and Koskimaa *et al*.^7^ and co-workers. Sarkola and coworkers confirmed the PCR positive HPV results by localizing the HPV16 and HPV6 DNA in syncytiotrophoblasts with tyramide amplified *in-situ* hybridization.

We recognized placental microbiota composed of *Firmicutes*, *Proteobacteria*, *Actinobacteria* and *Bacteroidetes*, which is in line with recent previous studies^17,19,20,38,39^. With HPV positive samples, we saw a significant increase in the amount of *Lactobacillus* genus which was mainly composed by *L*. *iners*, *L*. *crispatus*, *L*. *jensenii*, *L*. *gasseri* and *L*. *reuteri* species. Lactobacillus is the most abundant phyla in the vaginal microbiota^40^ but has also been detected in placenta in previous studies without analyzing the presence of HPV^19,38^. The most abundant Lactobacillus species in cervix and placenta were *L*. *iners* (41.0 vs. 0.2, p = 0.0000018), *L*. *crispatus* (13.8 vs. 0.1, p = 0.0012), *L*. *gasseri* (120.3 vs. 0.06, p = 0.023) and *L*. *jensenii* (6.01 vs. 0.0035, p = 0.042, respectively) without taking into account the HPV status of the samples.

In the present study, *Ureaplasma* spp. (*Ureaplasma parvum* 99% identity) was enriched in HPV positive placenta sample group. *Ureaplasma* spp. has previously in several studies been associated with chrorioamnionitis, which jeopardizes normal pregnancy and might lead to preterm delivery^38,41–45^. However, our results did not support the view that HPV presence would explain pregnancy complications and risk pregnancies as all had normal pregnancy without any complication. Nevertheless, the growing evidence of bacteria and virus in the placenta warrants further investigation from the point of view of a potential causal role in pregnancy complications including chorioamnionitis and preterm delivery^46^.

HPV infection has previously been linked to higher richness in vaginal microbiota^3,24–26,29,47^ and may be regarded as a predictor of imbalanced vaginal flora, especially decreased amount of *Lactobacillus*^23,27,47,48^. In the present study, we did not detect differences in the amount of *Lactobacillus* but rather an increase of *Peptostreptococcaceae*, *Enterococcaceae* and *Haemophilus* in HPV negative cervical samples. *Peptostreptococcus* has previously been associated with high-grade invasive cervical carcinoma when encountered together with HPV infection^29^. Interestingly, *Enterococcus* has also previously been linked to HPV-16 positive cervical cancer biopsies^49^ in contrast to our present study. Still, these previous findings have been made in single studies only and *Enterococcus* has even been observed from asymptomatic and healthy female vaginal flora^50^. It is currently not clear whether the vaginal microbiota play an important role in acquiring and persistence of HPV infection^51^. We have also previously presented data that some of the women from Finnish Family HPV Study, who presented HPV16 positive, developed incident cervical intraepithelial neoplasia within 14 years of follow-up^52^. Whether *Lactobacillus* plays a protective role in HPV clearance remains also to be determined^51,53,54^. Moreover, LEfSe test showed higher abundance of unclassified *Coriobacteriaceae* genus in HPV positive group cervical samples which was mostly attributable to *Atopobium vaginae*. *A*.*vaginae* has previously been reported to be associated with HPV persistence in cervico-vaginal region^27,51^ and bacterial vaginosis^55,56^. Still, *A*.*vaginae* has also been suggested in some cases to be a part of healthy vaginal microbiota^57–60^. This would suggest that present HPV infection and the observed changes in cervical bacterial microbiota may independently or synergistically create an environment favoring HPV persistence, dysbiosis and neoplastic transformation.

In this study, we observed a number of women with multiple HPV infection simultaneously at different body sites. Other studies have also observed this phenomenon but the cause behind this is currently not known^61–64^. HPV infection has primarily been considered as a sexually transmitted disease but recent research has begun to challenge this notion and so far we also know that vertical transmission during delivery or later in life, horizontal transmission and even autoinoculation have been established as possible ways of acquiring HPV infection^3,65^. Based on the results of the current study, we are able to conclude that bacterial microbiota can be one of the factors influencing the composition of overall microbiota in different anatomical sites.

The mouth harbors one of the most diverse microbiota in the human body and the most abundant bacterial phyla in the oral cavity include *Actionbacteria*, *Bacteroidetes*, *Firmicutes*, *Fusobacterium*, *Streptococcus*, *Veillonella* and *Proteobacteria*^40,66^. In our study the presence of HPV infection increased the amount of *Selenomonas* spp., *Megasphaera* spp. and *TM73 in* the oral microbiota. *Selenomonas* is a part of oral normal microbiota and oral biofilm but is also associated in disease, especially *Selenomonas noxia* in (aggressive) periodontitis (gum disease) and increased caries activity^67–69^. *Megasphaera* spp. is a part of dental plaque, so called early colonizer and has been discovered in endodontic abscess specimens^70^. The microbiota changes we found in oral HPV positive samples have not been earlier connected with HPV infection but may affect oral health. The association between oral HPV infection and oral health has not been extensively investigated but our data suggests that the oral microbiota composition in the presence of present HPV infection may favor potential pathogens.

Our study has several limitations. In our material of 329 mothers and deliveries and 315 placentas, only 13 placenta samples were HPV positive. This small number of HPV positive samples most likely explains why differences in the overall richness in the microbiota were not found. Secondly, the amount of usable DNA residues from bacteria in placenta samples is low which renders drawing definitive conclusions of the overall composition of microbiota difficult.

To our knowledge, this is the first study to show that HPV infection is associated with altered bacterial microbiota composition in the placenta and the mouth. We used the same protocol to indicate microbiota from oral, cervical and placenta samples. The diversity and composition of the bacterial microbiota was characterized by sequencing of the 16S rRNA gene and because a single bacterium can harbor multiple sequences of the 16S rRNA genes we have better possibilities to detect them as a sign that there has truly been a bacterial colonization. Still we were only able to investigate a small proportion of the placenta samples and needed to exclude some samples because the low number of reads to strengthen the reliability of our data. This definitely has an impact on our results but it has been an issue also in recent published articles investigating the placenta microbiota^18,19^.

In summary, we report a novel finding of association between microbiota and HPV infection. Our data may be interpreted to corroborate the hypothesis of a distinct microbiota of placenta. These initial findings still need to be replicated in larger studies to either confirm our findings or to reveal new perspectives into this neglected field of viral bacterial interactions. Whether the changes in bacterial microbiota predispose or result from HPV and if it influences HPV persistence, remains also to be determined in future studies.

## Methods

Samples collected in the Finnish Family HPV Study^71^ were utilized in this study. The original study was designed to evaluate the interactions of HPV infection in families. A total of 329 pregnant women in their third trimester were enrolled in the study together with their male spouses (n = 131) and offsprings to come (n = 313) with 6-year follow-up.

For the present substudy, DNA samples from the placenta, the uterine cervix and the mother’s mouth taken during the last trimester were included. The presence of HPV DNA has been examined earlier^7,12,62^. The subjects were selected according to the HPV status of the placenta and mode of delivery. Altogether 13 placentas with HPV DNA were available. Thirteen HPV DNA negative placentas from vaginal deliveries with 13 HPV DNA negative placentas from caesarean section deliveries were selected as controls. The placenta samples obtained after delivery included all the tissue layers from the maternal side of placenta and the HPV DNA was localized in syncytiotrophoblasts^12^. Furthermore, the oral and cervical samples of these women had been collected as mucosal scrapings with a brush (Cytobrush, MedScand, Malmö, Sweden) and immediately frozen −70 °C until used.

The clinical study and its amendments were found acceptable by the Ethical Committee of the Intermunicipal Hospital District of Southwest Finland (#3/1998, #2/2006, 45/180/2010). The methods were carried out in accordance with the relevant guidelines and regulations. Informed consent was obtained from all the subjects participating in the study.

### DNA extraction

HPV DNA was extracted by the high salt-method^72^.

### HPV detection

HPV testing was performed with nested PCR using MY09/MY11 as external primers and GP05+/GP06+ as internal primers followed by genotyping with Multimetrix® kit (Progen Biotechnik GmbH, Heidelberg, Germany), which detects 24 HPV types (low-risk HPV6, 11, 42, 43, 44, and 70; high-risk HPV16, 18, 26, 31, 33, 35, 39, 45, 51, 52, 53, 56, 58, 59, 66, 68, 73 and 82 types) as described in Koskimaa *et al*.^7^ and Syrjänen *et al*.^62^.

### 16S bacterial gene sequencing

Isolated DNA concentrations were measured using a Qubit® 2.0 Fluorometer (Life Technology, Carlsbad, CA, USA) and normalized to10 ng/μL. The V3-V4 region of 16S rDNA gene was amplified by PCR using Illumina adapter overhang nucleotide sequences following Illumina protocols. After 16S rDNA gene amplification, the multiplexing step was performed using Nextera XT Index Kit (Illumina, San Diego, CA, USA) and PCR product was checked in a Bioanalyzer DNA 1000 chip (Agilent Technologies, Santa Clara, CA, USA). Libraries were sequenced using a 2 × 300 pb paired-end run (MiSeq Reagent kit v3) on a MiSeq-Illumina platform (Lifesequencing sequencing service, Valencia, Spain). To rule out and control for possible contaminations, PCR amplification and libraries controls were also sequenced as negative controls.

### Bioinformatics and statistical analysis

Quality control of the FASTQ files was performed using Fastx tool kit version 0.0134 to remove reads with quality less than Q20, once the sequences were clean based on quality scores, we trimmed traces of the 16S rRNA primers and sequencing adapters using cutadapt version 1.2.5. After primer removal, sequences with <300 nucleotides read length were trimmed using perl scripting. Sequences were mapping against the human genome BWA version 0.7.1^73^ and filtered with samtools version 1.3.1–50. Clean FASTQ files were converted to FASTA files and UCHIME program version 4.2 was used to remove chimeras.

An open reference OTU picking method using 97% identity to the Greengenes 13_8 database was performed using QIIME pipeline (version 1.9.0)^74^. Singletons and OTUs with a relative frequency below 0.01 were removed. Sequences that could not be classified to domain level, or were classified as Cyanobacteria, Chloroplasts (as they likely represent cellulose and cotton material) and Rhizobiales (as potential environmental contaminants) were removed from the dataset.

Alpha diversity indices (Chao1: richness and Shannon: diversity) and beta diversity using UNIFRAC (phylogenetic) and Bray Curtis distance (non-phylogenetic) among samples were studied and PERMANOVA was used to test significance. Calypso software version 8.10 (http://cgenome.net/calypso/) was used with total sum normalization (TSS) for the statistical analysis, and also, Cumulative Sum Scaling normalization (CSS) for multivariate tests (Redundancy Analysis - RDA). Linear discriminant analysis effect size (LEfSe) was used to detect unique biomarkers (LDA score > 3.0) in relative abundance of bacterial taxonomy. P-values ≤ 0.05 were regarded as statistically significant.

### Data availability

The datasets generated during and/or analyzed during the current study are available from the corresponding author on reasonable request.

## Electronic supplementary material

Supplementary Tables 1 and 2
