# Supplementary material for: HPV infection and bacterial microbiota in the placenta, uterine cervix and oral mucosa
Source: Sci Rep. 2018 Jun 28;8:9787. doi: 10.1038/s41598-018-27980-3 (PMC6023934; doi:10.1038/s41598-018-27980-3)
Supplement: Supplementary file 1 — Supplementary Tables 1 and 2 [file 41598_2018_27980_MOESM1_ESM.pdf]

HPV infection and bacterial microbiota in the placenta, uterine cervix and oral mucosa

Heidi Tuominen[1,*], Samuli Rautava[2], Stina Syrjänen[1,3], Maria Carmen Collado[4], Jaana Rautava[1,3,]

[1]Department of Oral Pathology and Oral Radiology, Institute of Dentistry, Faculty of Medicine, University of Turku, Turku, Finland

[2] Department of Paediatrics, University of Turku & Turku University Hospital, Turku, Finland

[3] Department of Pathology, Turku University Hospital, Turku, Finland

[4] Department of Biotechnology, Institute of Agrochemistry and Food Science, Spanish National Research Council (IATA-CSIC), Valencia, Spain

[*]Corresponding author. Address: Department of Oral Pathology and Radiology, Institute of Dentistry, Faculty of Medicine, University of Turku, Lemminkäisenkatu 2, FIN-20520 Turku, Finland; e-mail address: heemtu@utu.fi

Supplementary Table 1. Taxonomical assignment of the reads obtained in the pooled negative controls.

**Phylum**	**Class**	**Order**	**Family**	**Genus**
p__Firmicutes	c__Bacilli	o__Bacillales	f__Planococcaceae	g__Lysinibacillus
p__Firmicutes	c__Bacilli	o__Lactobacillales	f__Streptococcaceae	g__Streptococcus
p__Proteobacteria	c__Betaproteobacteria	o__Burkholderiales	f__Comamonadaceae	g__
p__Proteobacteria	c__Betaproteobacteria	o__Burkholderiales	f__Comamonadaceae	g__Delftia
p__Proteobacteria	c__Betaproteobacteria	o__Burkholderiales	f__Oxalobacteraceae	g__Cupriavidus
p__Proteobacteria	c__Gammaproteobacteria	o__Pseudomonadales	f__Pseudomonadaceae	g__Pseudomonas

Supplementary Table 2: *Lactobacillus* species distribution in placenta and cervix.

OTU	Taxonomic (BLAST)	Cervix	Placenta
p__Firmicutes__g__Lactobacillus__s__iners_133075	*L.iners*	41,04 %	0,24%
p__Firmicutes__g__Lactobacillus_703741	*L.crispatus*	13,79%	0,12%
p__Firmicutes__g__Lactobacillus_581474	*L.gasseri*	10,28%	0,06%
p__Firmicutes__g__Lactobacillus_813944	*L.crispatus*	7,82%	0,1%
p__Firmicutes__g__Lactobacillus_463361	*L.jensenii*	6,01%	0,0035%
p__Firmicutes__g__Lactobacillus_574021	*L.gasseri*	0,25%	0,0035%
p__Firmicutes__g__Lactobacillus__s__reuteri_588197	*L.reuteri*	0,24%	0,001%
p__Firmicutes__g__Lactobacillus__s__iners_130468	*L.iners*	0,11%	0%
p__Firmicutes__g__Lactobacillus_134467	*L.crispatus*	0,11%	0%
p__Firmicutes__g__Lactobacillus__s__iners_New.ReferenceOTU30	*L.iners*	0,1%	0,001%
